# Prognosis related to staging systems for chronic lymphocytic leukemia

**DOI:** 10.1590/S1516-31802000000400002

**Published:** 2000-07-07

**Authors:** José Roberto de Faria, José Salvador Rodrigues de Oliveira, Rosa Malena Delbone de Faria, Maria Regina Regis Silva, Samuel Goihman, Miohoko Yamamoto, José Kerbauy

**Keywords:** Chronic Lymphocytic Leukemia, Prognosis, Clinical, Staging, Leucemia linfocítica crônica, Prognóstico, Clínica, Estadiamento

## Abstract

**CONTEXT:**

Chronic lymphocytic leukemia (CLL) is a clonal lymphoproliferative disorder, characterized by B lymphocytic proliferation. CLL is the most frequent adult leukemia in Western countries, accounting for 25 to 30% of all white leukemic patients.

**OBJECTIVE:**

To evaluate clinical and staging characteristics in prognosis of chronic lymphocytic leukemia.

**DESIGN:**

Evaluation of clinical-staging data.

**SETTING:**

Universidade Federal de São Paulo - Escola Paulista de Medicina / Universidade de Alfenas.

**SAMPLE:**

73 patients diagnosed from 1977 to 1994.

**MAIN MEASUREMENTS:**

Sex, ethnic origin, age, lymphadenopathy, splenomegaly, hepatomegaly, three or more areas of lymphoid enlargement, hemoglobin (g/dl), lymphocytes/mm^3^, Platelets/mm^3^

**RESULTS:**

Mean survival of patients was 76 months, median age was 65 years, ranging from 33 to 87. Forty-four patients (60.3%) were male and 29 (39.7%) female.

**CONCLUSION:**

The Binet system determined a better prognosis than Rai.

## RESUMO

**CONTEXTO:**

Diversas variáveis clínicas se relacionam ao prognóstico na leucemia linfocítica crônica; no entanto, o estadiamento de Binet determina melhor o prognóstico do que o estadiamento de Rai e Rai modificado.

**OBJETIVO:**

Avaliar características clínicas e laboratoriais no prognóstico da leucemia linfocítica crônica.

**TIPO DE ESTUDO:**

Estudos de correlação.

**PARTICIPANTES:**

73 patients diagnosticados entre 1977 e 1994.

**VARIÁVEIS ESTUDADAS:**

Sexo, origem étnica, idade, linfadenomegalia, esplenomegalia, hepatomegalia, três ou mais áreas com aumento linfóide, hemoglobina, linfocitos, plaquetas.

**RESULTADOS:**

A sobrevida média dos pacientes foi 76 meses, idade média de 65 anos, variando de 33 a 87 anos. Quarenta e quatro pacientes (60,3%) eram do sexo masculino e 29 (39,7%) do sexo feminino. A análise univariada mostrou que o nível de hemoglobina (P = 0,001), número de plaquetas (P = 0,03), linfocitose periférica (P = 0,03), número de áreas linfóides acometidas (P = 0,01), assim como os estadiamentos de Rai (P = 0,02), Rai modificado (0,007) e Binet (P = 0,003) relacionaram-se significantemente com a sobrevida. A análise multivariada, pela regressão de Cox, demonstrou que o sistema de Binet determina melhor o prognóstico do que os estadiamentos de Rai e Rai modificado.

**CONCLUSÃO:**

O estadiamento de Binet é melhor que o estadiamento de Rai e Rai modificado na determinação do prognóstico.

## INTRODUCTION

Chronic lymphocytic leukemia (CLL) is a clonal lymphoproliferative disorder, characterized by B lymphocytic proliferation.^1^ CLL is the most frequent adult leukemia in Western countries, accounting for 25 to 30% of all white leukemic patients.^2^ The mean age for the disease is greater than 50 years and the male to female ratio is 2:1.^3^ The illness seems to occur in 0.8 new cases per 100,000 persons per year in Brazil.^4^

Most patients are asymptomatic regarding anemia, lymph nodes, spleen and/or liver enlargement, as well as for hemorrhages and infective complications demanding medical attention.^1,5^ Rai et al.^6^ suggested that a persistent peripheral blood lymphocytic count greater than 15000 per mm^3^ and lymphocytic marrow cellularity greater than 40% are essential findings for making its diagnosis.

Many studies have been tried, since early in the 20th century, to determine prognostic factors and staging systems for predicting survival. These studies were consolidated into the criteria devised by Rai et al. (1975)^6^ and Binet et al. (1981).^7^

Rai's proposals consisted of 5 established steps: stage 0 - only lymphocytosis (in blood as well as in marrow); stage I - lymphocytosis associated with enhanced lymph nodes; stage II - lymphocytosis plus liver and/or spleen enlargement, with or without lymph node involvement; stage III - lymphocytosis in the presence of anemia, defined by hemoglobin levels below 11 g/dl; stage IV - lymphocytosis plus thrombocytopenia, evaluated by a platelet count of less than 100,000 per mm^3^.^6^

Binet et al.^7^ summarized the Rai system and recommended only three stages, as follows: stage A - presence of one or two lymphoid enlargements; B - three or more areas and C - presence of anemia confirmed by hemoglobin less than 10 g/dl or thrombocytopenia, with a platelet count of less than 100,000 per mm^3^.

The International Workshop on CLL (1981) recommended the association of Binet and Rai in order to analyze clinical and evolution data. The following stages were suggested: stage A(0), A(I) and A(II) as low risk; B(I), B(II) as intermediate and C(III) and C(IV) as high risk.^8^ However, in actual practice, this system was not completely acceptable worldwide, and clinicians have continued to use either the Binet or Rai methods.^9^ The National Cancer Institute (1996) proposed a modified Rai system as follows: low risk (Rai stage 0); intermediate risk (Rai stage I plus stage II) and high risk (Rai stage III plus IV).^9^ At the moment many other prognostic factors are being studied, particularly abnormal expression of oncogenes (Bcl-2, Bcl-X_L_, Bcl-W, Bax, Bak, Mcl-1, Bag, p-53, and others).^10^

Our intention here was to evaluate clinical and staging features in Brazilian CLL patients so as to understand their prognostic meaning better, by comparing the Binet, Rai, and modified Rai staging systems.

## METHODS

One hundred and two CLL patients were admitted into São Paulo Hospital and Alzira Velano Hospital between 1977 and 1994. The data for 26 of them were not consistent with what was on their medical registers, which might have allowed us to determine either their survival or clinical staging, and 3 other patients had misinterpreted diagnoses. The 73 remaining patients were included for final evaluation. All of these fulfilled Binet, Rai and modified Rai staging requirements.

All patients were submitted to conventional chemotherapy schedules with combinations of oral chlorambucil and prednisone for early stage disease or cyclophosphamide, vincristine, prednisone and adriamycin for advanced disease.

Diagnosis was based on history, physical examination, presence of more than 5.0 × 10^3^ lymphocytes per mm^3^ in peripheral blood, and at least 30% of lymphocytic marrow involvement in marrow aspiration analysis.^1^ The staging was obtained according to the Binet,^7^ Rai^6^ and modified Rai systems.^9^ Survival curves were obtained by limited extrapolation of the Kaplan & Meier method.^11^ The curves obtained were compared using the Wilcoxon and Cox-Mantel tests.^12^_._ Multivariate analyses were performed using multiple Cox regressions.^13,14^ "P" was considered to be significant when less than 0.05, for all tests. The statistical programs utilized were: KMSURV - Univariate Survival Data Analysis, May 89, Ludwig Institute for Cancer Research, São Paulo Branch - Epidemiology and Biostatistics Unit; BMDP - Biomedical Data Package - Survival Analysis with Covariates - Cox Models, May 84, Health Science Computing Facility, University of California (UCLA), Los Angeles.^14^

## RESULTS

There were 56 (76.7%) live patients at the end of our observation. Mean survival was 76.1 months, ranging from 1 to 140. Forty-four (60.3%) were male (M) and 29 (39.7%) female (F), with M/F ratio of 1.52. Mean age was 63.9 years, ranging from 33 to 87. Mean survival after the age of 65 years was 78.5 months versus 66.9 before this (P = 0.55). Females had longer survival than males, but not reaching statistical significance (P = 0.09). Table 1 shows the univariate analysis results. To better compare patients in Binet stages A and B, all those with anemia and thrombocytopenia (stage C) were excluded for separated analysis (Table 2). Mean survival according to the Rai, modified Rai and Binet staging systems is summarized in Table 3.

**Table 1:** Results of the univariate analysis

Variable	Category	Number (%)	Mean Survival (months)	P
Sex	Males	44 (60.3%)	61.13	0.09
	Females	29 (39.7%)	87.76	
Ethnic origin	Whites	60 (82.2%)	74.12	0.72
	Non-whites	13 (17.8%)	65.15	
Age	> 65	37 (50.7%)	78.50	0.55
	< 65	36 (49.3%)	66.91	
Lymphadenopathy	Present	40 (54.8%)	53.67	0.27
	Absent	33 (45.2%)	82.84	
Splenomegaly	Present	29 (39.7%)	58.88	0.07
	Absent	44 (60.3%)	124.12	
Hepatomegaly	Present	24 (32.9%)	63.33	0.23
	Absent	49 (67.1%)	120.16	
Three or more areas of	Present	17 (23.3%)	42.47	0.01
lymphoid enlargement	Absent	56 (76.7%)	90.54	
Hemoglobin (g/dl)	≥ 10	47 (64.4%)	94.07	0.001
	< 10	26 (35.6%)	33.74	
Hemoglobin (g/dl)	≥ 11	39 (53.4%)	94.72	0.004
	< 11	34 (46.6%)	36.94	
Hemoglobin (g/dl)	≤ 10	30 (41.1%)	36.03	0.02
	< 12 and ≥ 10	20 (27.4%)	67.82	
	≥ 12	23 (31.5%)	98.49	
Lymphocytes / mm^3^	≥ 36,000	37 (50.7%)	54.23	0.03
	< 36,000	36 (49.3%)	89.46	
Platelets / mm^3^	≥ 100,000	58 (79.5%)	88.55	0.03
	< 100,000	15 (20.5%)	36.4	

**Table 2:** Mean survival (months) of patients considering clinical variables except anemia and thrombocytopenia

Variable	Present	Absent	P
Lymphadenopathy	56.49	115.25	0.04
Splenomegaly	73.92	140.00	0.02
Hepatomegaly	79.26	140.00	0.06
Three or more areas of lymphoid enlargement	52.05	115.25	0.01

**Table 3:** Mean Survival (months) according to the Rai, Binet and modified Rai staging systems

Stage	Number (%)	Survival	P
Rai			
O*	11 (15.0)	140.00	0.02
I*	6 (08.2)	27.00	
II	26 (35.6)	79.82	
III	14 (19.1)	13.17	
IV	16 (21.9)	36.46	
Binet			
A	29 (40.7%)	115.25	0.003
B	14 (19.2%)	52.05	
C	30 (41.1%)	31.01	
Modified Rai			
Low risk*	11 (15.0%)	140.00	0.007
Intermediate risk	32 (43.9%)	83.34	
High risk	30 (41.1%)	32.01	

* All patients were alive at the time of cutoff.

### Univariate and multivariate analysis

**Figure 1:**
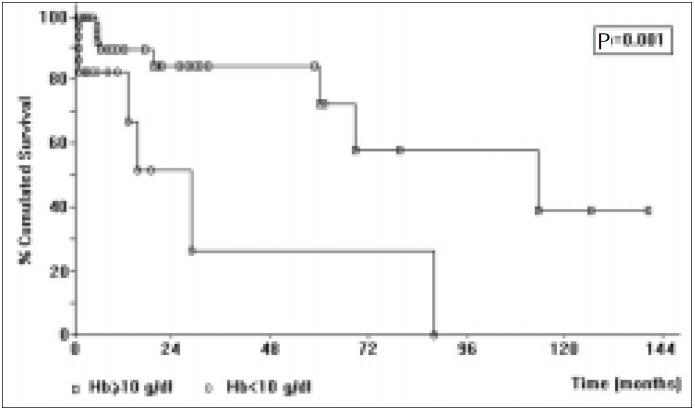
Survival of CLL patients according to hemoglobin (Hb) level.

**Figure 2:**
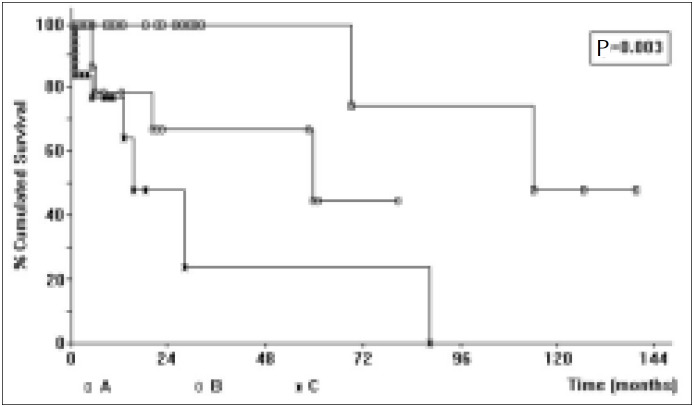
Survival of CLL patients according to Binet staging system.^7^

Seven variables were compared with prognosis (survival): 1) hemoglobin levels with cutoffs of 12, 11 and 10 g/dl, with P = 0.02, 0.004 and 0.001, respectively (Figure 1); 2) Binet staging system (P = 0.003; Figure 2); 3) Rai staging system (P = 0.02); 4) Modified Rai staging system (P = 0.007); 5) Number of lymphoid enhancement areas (P = 0.01); 6) Platelet count, with a cutoff of 100,000 per mm^3^ (P = 0.03); and 7) Number of lymphocytes in peripheral blood, greater than 36,000 per mm^3^ (P = 0.03). However, multivariate analysis only showed a significant independent effect on survival for the Binet system.

## DISCUSSION

Since the Minot & Isaacs study,^15^ many other studies have been carried out with the purpose of better establishing the prognosis of patients with CLL. Rai and Binet reported useful concepts but, so far, no paper has explained the heterogeneity in the prognosis of CLL.^16^

We began our study with the analysis of patients’ sex. There is agreement in the literature that the incidence is higher in men than in women, with the male/female ratio being about 2:1.^17^ Our cases showed a 1.52/1.00 ratio, which confirms the same tendency. Reports on the importance of patients’ sex on the prognosis are also heterogeneous in their results. Many authors have reported a better prognosis in women when compared to men,^18-22^ while others have not found a significant difference between the sexes.^23-26^ Our findings coincide with those of the latter group. Although we found higher mean survival among females, the difference was not significant.

Patients’ ages at the time of diagnosis most frequently range between the 6th and 7th decades of life.^27^ The average age in our cases was 63.9 years, with a median of 65 years, similar to the literature. The relationship of age to prognosis is not homogeneous in the literature. Minot & Isaacs^15^ and Paolini et al.^20^ correlated low age with a bad prognosis, but Boggs et al.,^23^ Hansen^18^ and Lee et al.^21^ reported the opposite. Although we found a higher mean survival in patients aged over 65 years, age differences were not statistically significant in our study (P = 0.55).

The incidence of CLL in Africa seems to be lower when we compare it with the incidence in white men. According to Linet & Cartwright,^27^ this fact is due not to low incidence among black people, but to the lack of diagnosis. We consider that it is not advisable to analyze prognosis in relation to race in Brazil, in view of the great interracial mixing in this country. We found a higher incidence in white patients (82.2%). However, this does not permit us to conclude that there is a genuinely low incidence in black people. The skin color was not significant in survival (P = 0.72).

No difference was found in our study regarding the mean of patients’ survival when we analyzed the presence of adenomegaly, splenomegaly and hepatomegaly (Table 1). Nevertheless, when we analyzed the presence of more than 3 areas of infiltration, there was a significant relation with survival (P = 0.01).

As there was a significant difference in the survival of patients in Binet's stages A and B, we performed the analyses of the same clinical variables, excluding the patients with anemia and/or thrombocytopenia, to avoid the effect of these latter variables on the prognosis. Thus, in patients without anemia and/or thrombocytopenia, we observed that the presence of both adenomegaly (P = 0.04) and splenomegaly (P = 0.02) caused a lower survival. Hepatomegaly showed lesser importance in the determination of survival (P = 0.06) and the number of affected areas was the most statistically significant parameter (P = 0.01). These findings permit us to conclude that prognosis really changes with the extent of the disease (Table 2).

Among the clinical variables, hemoglobin exhibited the most significant relationship with survival. According to various authors,^6,7,15,18,23,24,28^ hemoglobin is one of the most important variables in the survival of patients with CLL, and our results agree with those in the literature. We first analyzed hemoglobin at two cutoff levels (10 g/dl and 11 g/dl). As we found a significant relationship with survival at both levels (P = 0.001 and 0.004, respectively), we subdivided the hemoglobin levels into three groups (Hb = 10 g/dl, 10 g/dl < Hb < 12 g/dl and Hb = 12 g/dl). Even with the subdivision into three groups, the hemoglobin levels kept a significant relationship with survival (P = 0.006), showing a mean survival time of 36.20 months, 68.45 months, and 98.49 months, respectively. We have thus demonstrated that, as the levels of hemoglobin decrease, the patients’ mean survival time also decreases. The hemoglobin threshold for the determination of anemia was arbitrarily chosen as 10 g/dl in Binet's staging and 11 g/dl in Rai's staging. Hence, we think that patients with C(III) staging should not be considered in relation only to the presence of anemia, but should also be evaluated for hemoglobin level as a prognosis determinant, as progressively lower hemoglobin values lead to progressively worse prognoses (Table 1, Figure 1).

A platelet count lower than 100,000 per mm^3^ (P = 0.02) represented a significant prognosis, as well as a lymphocyte count of greater than 36,000 per mm^3^ (P = 0.03). With regard to platelet count, our results agree with those of Rai et al.,^6^ but disagree with others.^21,31-33^ For the latter, anemia should be a more relevant factor than thrombocytopenia. The prognostic significance of the lymphocyte count is controversial.^15,18,19.24,25,29-31^ In our observations, with cutoffs at 50,000/mm^3^, 100,000/mm^3^, and 150,000/mm^3^, we found no significant relationship with survival, probably because patients with more than 36,000 per mm^3^ may present a worse prognosis, as was also observed by Baccarani et al.^30^

We found that the three most important clinical variables for prognosis of CLL patients are hemoglobin with a cutoff at 10 g/dl (P = 0.001); number of lymphoid infiltration areas (P = 0.01), showing our agreement with the parameters for determination of groups A, B and C of the Binet staging system;^7^ and peripheral lymphocytosis (P = 0.03).

After evaluating our patients in accordance with Rai's staging, our findings agree with those in the literature. There was a significant difference in survival in our groups, but it did not decrease from stage 0 to stage IV. Survival was shorter in stage I than in stage II, but it may have occurred due to the sample size (6 patients) and a briefer follow-up. Moreover, all of them were alive at the end of this study. Survival in stage IV was not shorter than in stage III, which suggested that the platelet count may not have significant importance in the prognosis of CLL.

Although classic, the Rai system subdivides the disease into 5 stages, making the treatment approach more difficult due to the great number of subgroups. Moreover, Rai considers that liver and spleen infiltration occurs later than lymph node enlargement. Some authors^18,23,28^ have not infrequently observed cases of marked splenomegaly in the absence of adenomegaly. The modified Rai system is similar to the Binet system and was significant in the univariate analysis (P = 0.007), but despite the small number of groups the multivariate analysis showed that the Binet method expresses a better prognosis of CLL. We have also demonstrated that both the hemoglobin level and the number of lymphoid areas involved, as seen by Binet et al.,^7^ correlate with survival in the univariate analysis. However, only the Binet system remained significant in the multivariate analysis, being a simpler and more accurate method for evaluating the prognosis of CLL.

## CONCLUSIONS

The significant variables in the univariate analysis were: number of areas with enlarged lymphoid, hemoglobin, platelets, peripheral lymphocytosis and Rai, modified Rai and Binet systems. Multivariate analysis showed Binet staging is better than Rai and modified Rai for evaluating prognosis.
